# Ion release from three different dental alloys – effect of dynamic loading and toxicity of released elements

**DOI:** 10.1080/26415275.2020.1747471

**Published:** 2020-04-24

**Authors:** Ketil Hegerstrøm Haugli, Morten Syverud, Jan Tore Samuelsen

**Affiliations:** a Department of Life Sciences and Health, Oslo Metropolitan University, Oslo, Norway; b Nordic Institute of Dental Materials (NIOM), Oslo, Norway

**Keywords:** Dental alloys, corrosion, cytotoxicity

## Abstract

**Objective:**

The aims of this *in vitro* study were to assess if dynamic loading increases the metal ion release of selected dental alloys and to evaluate the cytotoxicity of the released metal ions.

**Materials and methods:**

One Pd–Ag alloy (Aurolite 2B) and two Co–Cr alloys (Wirobond 280 and d.Sign 30) were investigated. Two different corrosion immersion tests were used: a standardized static test (ISO 22674: 2016) and an experimental dynamic test. Both tests involved immersion of the specimens in a lactic acidic solution (pH = 2.3). Inductively coupled plasma mass spectrometry was used to identify and quantify released elements. A human monocyte cell-line (THP-1) was exposed to serially diluted solutions containing the selected metal ions. Cell viability was measured using the methyl-thiazolyl-tetrazolium assay.

**Results:**

According to the threshold defined in ISO 22674, only low concentrations of released elements were observed for both corrosion tests. No increase in metal ion release from the dynamic test compared with the static test was observed. Of the released elements, only Zn(II) and Co(II) showed a cytotoxic effect on THP-1 cells at 250 µM and higher concentrations. No increased viability loss was observed when adding other released elements to the exposure mixture.

**Conclusions:**

The tested alloys showed low levels of metal ion release from both static and dynamic corrosion testing. Dynamic loading did not increase the metal ion release compared to the static corrosion test. Concentrations of 250 µM and above of Zn(II) and Co(II) showed a cytotoxic effect on THP-1 cells.

## Introduction

Patient safety is of considerable concern when selecting dental materials, controversy remains around biocompatibility. Although metal–ceramic restorations are among the most frequently used dental restorative alternatives for replacing single or multiple teeth, and implant-supported prostheses, the evidence surround biocompatibility cannot keep up with the increase in the selection of metal–ceramic alloys available [1]. Relevant factors for biological response to an alloy depends on the biological effects of released elements, the quantities of released elements and the duration of tissue exposure to these elements [2]. It is evident that all dental alloys release elements and from a biocompatibility standpoint, quantification and identification of the released elements is regarded the most relevant measure of corrosion [3]. Released elements react to form compounds, and these compounds can interact locally or systemically increasing the risk of adverse effects [4]. From these perceptions, the biological safety of metal–ceramic alloys is of great concern as a public health issue.

The current standard for dental alloys (ISO 22674: 2016) [5] classifies alloys according to their mechanical properties rather than the composition. Knowledge about alloy composition is important in the selection of alloys and assessments regarding the biocompatibility. The current ADA (American Dental Association) specification classifies dental alloys by composition, dividing alloys into three groups: (1) *high noble*, with a noble-metal content of at least 60 wt% and a gold content of at least 40%; (2) *noble*, with a noble-metal content at least 25% (no stipulation for gold); and (3) *predominately base metal*, with a noble-metal content less than 25% [6]. The terms stated in the ADA specification will be used in this paper. High noble alloys have been the material of choice for many years [7]. Although they show good corrosion resistance and acceptable mechanical properties, the increasing cost of gold has motivated the search for alternative alloys. However, these alternative noble and predominantly base metal alloys may be less resistant to corrosion [8]. Predominantly base metal alloys, such as cobalt–chromium (Co–Cr) and titanium-based alloys, are increasingly used in dental restorations due to good mechanical properties, effective manufacturing from computer-aided design and computer-aided manufacturing (CAD/CAM) production and alleged acceptable biocompatibility. The use of advanced CAD/CAM technology in the production of alloy based dental restorations can be performed either from milling (subtractive manufacturing) or 3 D printing (additive manufacturing) [9]. Although novel, CAD/CAM manufacturing techniques are applied in the dental profession, however the manufacturing of Co–Cr based metal–ceramic restorations from conventional casting technique is still commonly used [10]. High initial investment cost is limiting the adoption of CAD/CAM technology to small-medium sized dental laboratories [11]. Therefore, fixed metal-ceramic prostheses in high noble and noble alloys continue to be manufactured with conventional casting techniques.

The oral environment is a hostile environment regarding corrosion. Factors affecting corrosion in an individual are: salivary pH, intake of food or pharmaceuticals, temperature changes, bacterial plaque formation, and changes of oral health status and stress [12]. Also, the corrosion depends largely on the physical properties of the alloy (e.g. composition, microstructure and surface state of different elements) [13]. Since intraoral corrosion is multifactorial, the corrosive behaviour of dental alloys is difficult to predict, and consequently challenging to determine the biocompatibility. The ISO standard for testing metallic materials used in fixed prostheses (ISO 22674) implies that the total release of metal elements from an alloy specimen in a lactic acidic solution shall not exceed 200 µg/cm^2^ after a 7-day test period. This standard uses the term ‘corrosion resistance’ to describe metal ion release [5]. However, there are factors that are not considered in the standard. Firstly, masticatory forces introduce stresses to dental alloys, which further could increase corrosion. Secondly, the widely differing toxic potential of the released elements is not taken into account, only the total amount of released elements. Therefore, this study applies a method for simulating dynamic (cyclic) stresses from mastication and evaluate the effect on corrosion. Since metal–ceramic restorations are preferred in the posterior region where masticatory forces are high [10], an approach to simulate such an environment was introduced.

Biocompatibility studies of dental alloys have been given much attention over the past two decades [1,3,4,12]. However, there is a lack of studies focusing on the effect of combined toxicity of released elements on established cell-lines. In this *in vitro* study, a human monocyte cell-line (THP-1 cells) was exposed to serially diluted combinations of elements that were released from the investigated metal–ceramic alloys in addition to cytotoxic evaluation of the individual elements. The MTT (3-(4, 5-dimethylthiazolyl-2)-2, 5-diphenyltetrazolium bromide) assay was used for cytotoxicity evaluation.

The primary aim of this study was to assess the metal ion release of one noble Pd–Ag alloy and two Co–Cr-based alloys and to compare the standard static corrosion test with an experimental dynamic corrosion test. A second aim was to evaluate the cytotoxicity of the released elements, both individually and in combinations.

## Materials and methods

### Assessment of metal ion release

#### Preparation of alloy specimens

Three specimens from each alloy (*n* = 3) were designed in burnout wax pattern (Kerr casting wax sheet, KaVo Kerr, CA, USA) with dimensions of approximately (34 × 13 × 1.5) mm, invested (Bellavest SH, Bego, Bremen, Germany) and cast. Table 1 shows the technical information of the Pd–Ag alloy, the two Co–Cr alloys and heat treatment specifications. The Pd–Ag specimens were cast in a vacuum-pressure machine (Combilabor CL-G; Heraeus, Hanau, Germany) while the two Co–Cr alloys were cast in an induction-heated air-pressure machine (Heracast IQ; Heraeus, Hanau, Germany). All specimens were sandblasted with 110 µm Al_2_O_3_ particles (Korox 110, Bego, Bremen, Germany) at 2–3 bar pressure to remove investment. At least 0.1 mm from the alloy surface was removed by grinding with wet silicon carbide grinding paper discs (SiC grinding paper, Struers, Ballerup, Denmark). After heat treatment, the oxidation film formed was removed using wet silicon carbide grinding paper discs with 4000 grit size. Finally, the specimens were ultrasonically cleaned in ethanol solution for 2 min, rinsed with distilled water and heat dried using a conventional hair dryer.

**Table 1.** Comparison of investigated alloys, their compositions and recommended heat treatment specifications.

Alloy type	Trade name, manufacturer, LOT No.	Composition in weight-%	Heat treatment (°C) Oxidation under vacuum/ 4 × ceramic firing under vacuum.
Base metal Co–Cr	Wirobond 280 BEGO, Bremen, Germany. LOT: WBA12753	Co: 60.2 Cr: 25 W: 6.2 Mo: 4.8 Ga: 2.9 Mn < 1	No (not recommended)/ Yes (*970)
Base metal Co–Cr	d.Sign 30 Ivoclar Vivadent, Schaan, Liechtenstein. LOT: $RR54002AQ	Co: 60.2 Cr: 30.1 Ga: 3.9, Nb: 3.2 Al, Fe, Li, Mo < 1	Yes (925)/ Yes (*970)
Noble Pd–Ag	Aurolite 2B Aurium Research U.S.A., San Diego, CA, USA. LOT: 6391202	Pd: 59.9 Ag: 26.3 In: 5 Sn: 5 Zn: 2 Au: 1.7 Ru < 1	Yes (1040)/ Yes (*970)

The heat treatment simulation procedures were carried out according to ISO 22674: 2016.

* Highest allowed temperature for simulating ceramics fusing to alloy.

#### Static immersion test

The static immersion test was performed according to ISO 10271: 2011 [14]. The specimens were placed in individual inert test tubes and immersed in lactic acidic solution (0,1 mol/l of lactic acid and 0,1 mol/l NaCl, pH = 2.3) for 7 d at 37 °C. The ratio between volume of the corrosion solution and surface area of a specimen were kept constant (1 ml solution per 1 cm^2^ of sample surface area). Subsequently, the aqueous solutions were transferred to individual polypropylene containers and sent for analysation. The elements in the solutions were analysed using an inductively coupled plasma mass spectrometer (ICP-MS) (PerkinElmer SCIEX ELAN DRC II; Waltham, MA, USA) conducted by a subcontractor (Fürst Medical Laboratory, Oslo, Norway).

#### Dynamic immersion test

**Figure 1.**
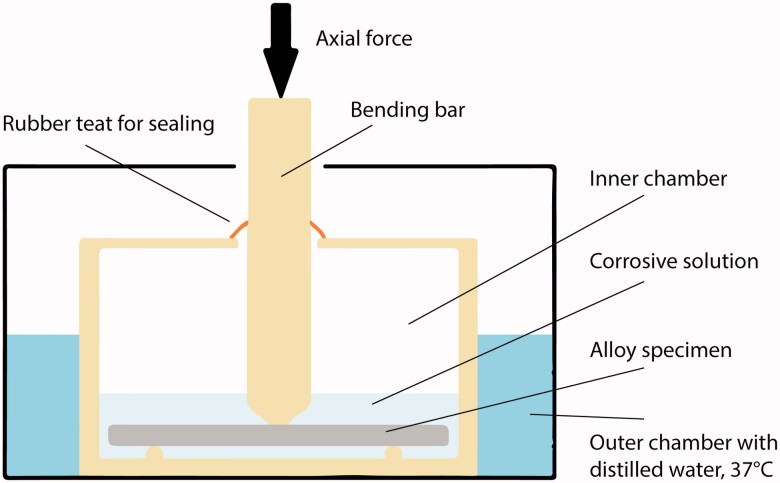
The dynamic corrosion test set-up. A specimen was placed on supporting pins immersed in artificial saliva (corrosive solution, pH= 2.3). The inner chamber, the bending bar and the supporting pins comprise of polyoxymethylene (POM) thermoplastic material. The inner chamber was sealed to prevent evaporation of the corrosive solution. The outer chamber was filled with distilled water set to constant temperature of 37 °C.

**Figure 2.**
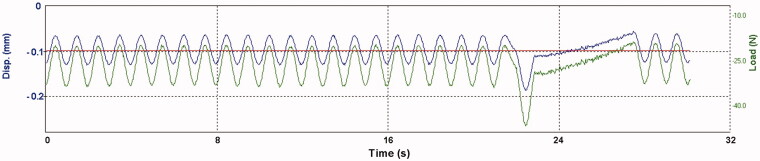
Load, displacement (y-axis) and time (x-axis) during cyclic loading of an alloy specimen. The green line shows force (N) applied to a specimen and the blue line shows the corresponding displacement as the alloy deflects. Each minima peak presents the highest values in force and displacement as a result from downward direction. Notice the highest force applied at the last cycle in a series.

The test setup is depicted in Figure 1. The size of the specimens from the static corrosion test were modified to approximately (32 × 6×1) mm to fit the dynamic loading machine (Electroforce 3330 Test Instrument; BOSE corp., Eden Prairie, MN, USA). Surfaces of all specimens were re-grounded and ultrasonically cleaned (as previously described) before mounting. Heat treatment was not repeated as this may change the metallurgical structure. The ratio between volume of the corrosion solution and surface area of a specimen was kept constant (same as for static immersion test). Cycles for loading and displacement were implemented in corresponding software (Wintest 7, Ver. 7.1; BOSE corp, Eden Prairie, MN, USA). The cyclic loading machine was set to perform a series of 40 load cycles at 1 Hz. The bending bar accounted for a pre-load of approximately 20 N. The first 39 cycles were set to a constant load depending on alloy stiffness (20-40 N). Deflection of the specimens were within the range of elasticity. For every 40^th^ cycle, a higher load, also within the elastic range (max. 70 N), was applied. Then, the cycle process was repeated. Figure 2 shows the parameters load (N, green line) and displacement (mm, blue line) relative to time (s) during cyclic loading of a specimen. The test was carried out for 7 d at 37 °C in lactic acid solution, pH = 2.3. The total number of cycles performed was approximately 550.000. Subsequently, the aqueous solutions were transferred to individual polypropylene containers for the assessment of released elements as described under ‘static immersion test’.

### Cytotoxicity tests

#### Selection of elements from compounds

Selection of elements for cytotoxicity testing were based on the results from the corrosion tests. Compounds with chloride as counter-ion were chosen based on comparison of different zinc compounds (Table 2). Due to low solubility of molybdenum chloride salts, MoO_2_ was selected as the preferred compound.

**Table 2.** Summary of selected compounds for cytotoxicity testing.

Chemical compound	Chemical formula	MW (g/mol)
Chromium(III) chloride hexahydrate	CrCl_3_ × 6H_2_O	266.45
Cobalt(II) chloride hexahydrate	CoCl_2_ × 6H_2_O	237.93
Indium(III) chloride	InCl_3_	221.18
Molybdenum(IV) oxide	MoO_2_	127.94
Niobium(V) chloride	NbCl_5_	270.17
Tin(II) chloride dihydrate	SnCl_2_ × 2H_2_O	225.65
Zinc chloride	ZnCl_2_	136.28
Zinc nitrate hexahydrate	Zn(NO_3_)_2_ × 6H_2_O	297.47
Zinc sulphate heptahydrate	ZnSO_4_ × 7H_2_O	287.54

#### Cells and cell culture treatment

THP-1 cells, a human leukemic cell line with monocytic and immunological functions, was purchased from the European Tissue Type Culture Collection (ECACC; Sigma-Aldrich, Halstadt, Germany). The cells were cultured in a 75-cm^2^ flask containing 20 ml RPMI 1640 media (Lonza, Rockland, ME, USA) including supplements (5 ml, 1 M HEPES-buffer, 5 ml Na-pyruvate, 2,75 ml gentamicin and 50 ml foetal bovine serum). Incubation was carried out in 5% CO_2_ and 37 °C. The cells were seeded at a concentration of 5 × 105 cells/ml. After 24-h seeding, the cells were prepared for cytotoxicity testing.

#### Cell exposure and MTT cytotoxicity tests

The MTT assay was performed as described in ISO 10993-5 [15]. Initially, cytotoxicity assessment of single element exposure to the cells were conducted. Each of the selected compounds were serially diluted (500 µM, 250 µM, 125 µM, 61 µM, and 31 µM). The control group was unexposed cells. Secondly, a combined exposure study including Co(II) and Zn(II) at a constant concentration of 125 µM (control group in the combined exposure study) were added to the same serial dilution setup as in the single element exposure test. Since a concentration of 125 µM for both Co(II) and Zn(II) indicated a threshold concentration where the viability still where within 70%, this value were chosen for the combined exposure study. Zn(II) was exposed together with In and Sn. Co(II) was exposed together with Cr, Mo and Nb. According to ISO 10993-5, a sample is considered to have a cytotoxic potential if the viability is reduced to less than 70% [15].

After 24-h exposure time, the MTT (3-(4,5-dimethylthiazol-2-yl)−2,5-diphenyl tetrazolium bromide) assay was used to evaluate cytotoxic potential [15]. Cleavage of the tetrazolium salt MTT into a blue-coloured product (formazan) is dependent on the activity of the mitochondrial enzyme, succinate dehydrogenase (SDH) [16]. In short, 300 µl MTT solution (0.5 mg/ml of MTT in PBS) was added to each well containing unexposed and exposed cells. After incubation for 1 h at 37 °C, the MTT solution was removed by centrifugation. DMSO was added to the cell pellet to dissolve the formazan product. Absorption at 570 nm was measured with a plate reader (Synergy H1; BioTek Instruments, Winooski, VT, USA). Recorded values were used as a measure of cell viability for each sample. The controls were set to 100% viability.

## Statistical analysis

To compare the ion release from static and dynamic test, the Student´s *t*-test was used.

MTT data were analysed with one-way ANOVA and Bonferronís multiple comparison test using GraphPad Prism 6 (GraphPad; La Jolla, CA, USA). Results were calculated as mean ± standard deviation (SD) of cell viability. P-values *p* < .05 were considered significant.

## Results

The results from the corrosion tests and the cytotoxicity tests are presented below.

### The released ions after corrosion tests

Table 3 shows the results from the *in vitro* static and dynamic corrosion tests of the alloys. The alloys showed good corrosion resistance (low metal ion release) compared to the threshold value of 200 µg/cm^2^ as stated in ISO 22674. Dynamic loading showed less element release compared to the static test for Aurolite 2B and Wirobond 280. A slightly higher release rate for dynamic loading compared to static test was observed for the d.sign 30 alloy, however the total elemental release was very low in both tests.

**Table 3.** Results from the static and dynamic corrosion immersion test analysed by ICP-MS. Mean values (of *n* = 3) are shown (µg released/cm^2^)±SD (except “#” where ± refers to max/min values; *n* = 2).

	Aurolite 2B		Wirobond 280		d.Sign 30	
Elements	Static	^#^Dynamic	Static	Dynamic	Static	Dynamic
Ag	0.11 ± 0.00	0.15 ± 0.00	–	–	–	–
Al	0.02 ± 0.00	0.02 ± 0.02	–	0.08 ± 0.04	0.01 ± 0.01	0.39 ± 0.33
B	0.09 ± 0.08	0.35 ± 0.19	0.04 ± 0.06	0.06 ± 0.01	0.03 ± 0.04	0.09 ± 0.05
*Co	–	–	15.13 ± 7.18	2.88 ± 2.70	0.49 ± 0.06	0.45 ± 0.11
*Cr	–	–	0.83 ± 0.34	0.81 ± 1.08	0.07 ± 0.01	0.08 ± 0.02
Fe	0.02 ± 0.01	0.02 ± 0.00	0.01 ± 0.01	0.04 ± 0.03	–	0.37 ± 0.48
Ga	–	–	0.42 ± 0.19	0.17 ± 0.19	0.02 ± 0.00	0.02 ± 0.00
*In	2.97 ± 0.23	0.07 ± 0.02	–	–	–	–
Mn	–	–	0.04 ± 0.02	0.01 ± 0.01	–	0.01 ± 0.00
*Mo	–	–	1.79 ± 0.60	0.38 ± 0.28	0.01 ± 0.00	0.01 ± 0.01
*Nb	–	–	–	–	0.14 ± 0.03	0.08 ± 0.05
Ni	–	–	–	0.01 ± 0.00	–	0.01 ± 0.01
Pd	–	0.32 ± 0.00	–	–	–	–
Ru	0.01 ± 0.00	–	–	–	–	–
*Sn	1.88 ± 0.31	0.06 ± 0.00	–	–	–	0.01 ± 0.01
*W	–	–	2.12 ± 0.81	0.47 ± 0.41	–	0.01 ± 0.02
*Zn	9.27 ± 8.34	0.89 ± 0.93	–	0.31 ± 0.31	–	0.43 ± 0.23
Total	14.37 ± 8.08	1.88 ± 0.80	20.38 ± 9.14	5.22 ± 5.04	0.77 ± 0.06	1.96 ± 1.17

Values less than 0.01 µg/cm^2^ are shown as “-“. Au, Be, Li and Cd were analysed but below the detection limit. No significant difference between static and dynamic test was measured for d.Sign (*p* = 0.15) and Wirobond 280 (*p* = 0.07). No statistics is performed on Aurolite 2B. None of the measurements suggest higher ion release in the dynamic test compared to the standard static test.

Elements marked with asterisk (*) were chosen for further toxicity testing. (#) Technical fault during run of one sample, thus *n* = 2.

### Cytotoxicity tests

**Figure 3.**
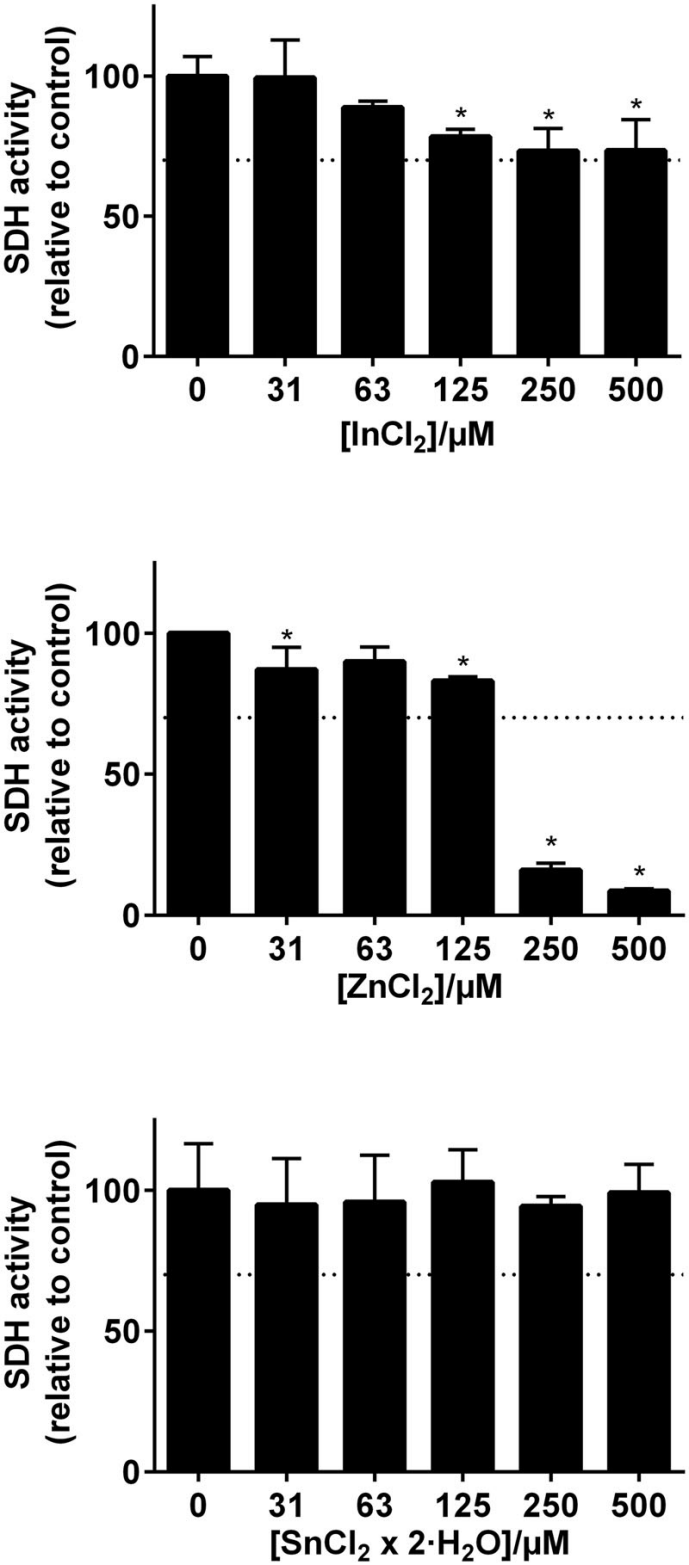
MTT-test results from the metal elements representing the Pd–Ag alloy (Aurolite© 2B) (*n* = 3, error bars indicate SD). *Statistical difference (*p* < .05).

**Figure 4.**
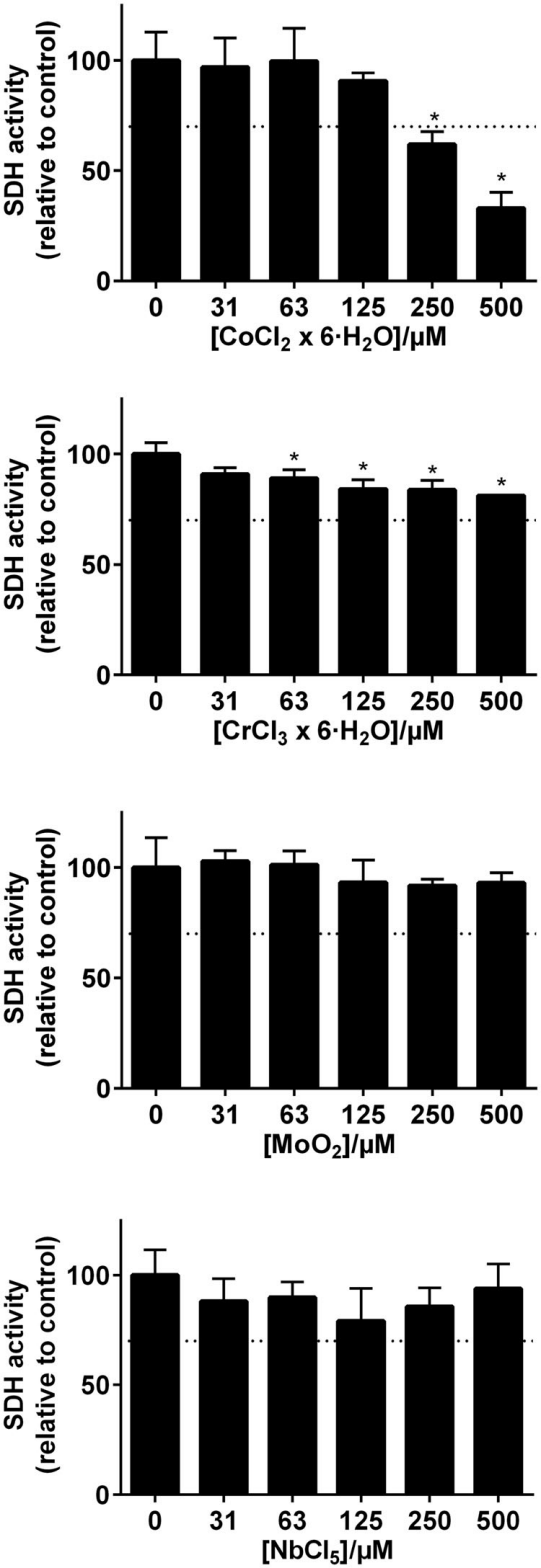
MTT-test results from the metal elements representing the Co–Cr based alloys (Wirobond© 280 and d.Sign© 30) (*n* = 3, error bars indicate SD). *Statistical difference (*p* < .05).

**Figure 5.**
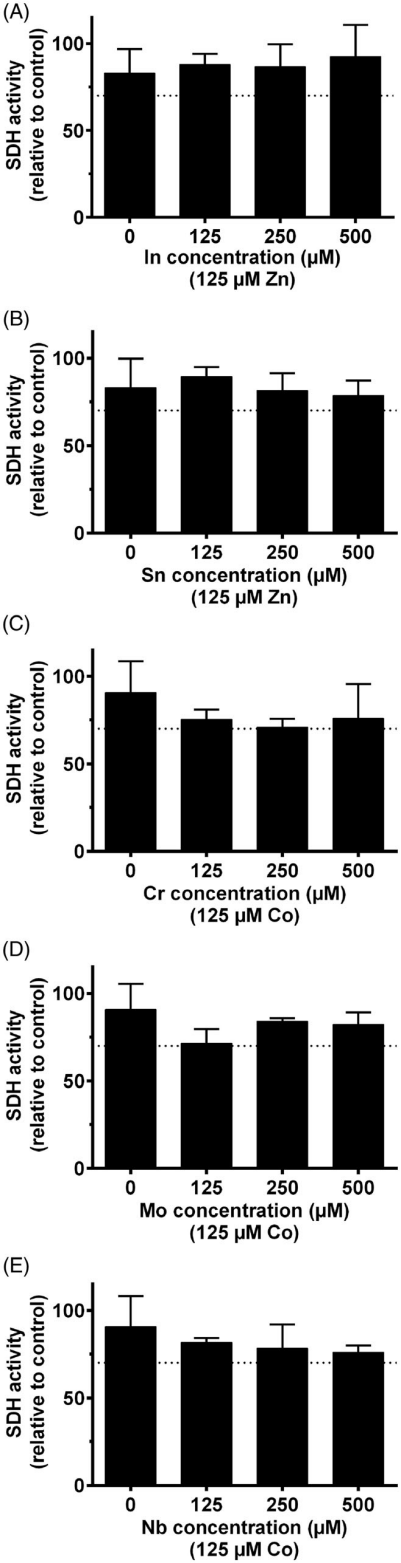
MTT-test results of elements combined. 125 µm Zn was combined with different concentration of In and Sn, representing the Pd–Ag alloy. For the Co–Cr alloys, 125 µm Co was combined with different concentrations of Cr, Mo and Nb.

Although some elements showed statistical significance of reduced viability, only Zn(II) and Co(II) reduced the number of viable THP-1 cells (measured as total SDH activity) to less than 70% at concentration levels above 250 µM (Figures 3 and 4). In the combined exposure studies, exposure of the cells to 125 µM Zn(II) in combination with In and Sn did not increase the toxicity compared to Zn(II) alone. Likewise, 125 µM Co(II) exposure in combination with Cr, Mo and Nb did not increase the toxicity compared to Co(II) alone (Figure 5).

## Discussion

Co–Cr and Pd–Ag alloys are regularly used in fixed prosthodontics [10,17,18]. In addition to the focus on physical properties of dental alloys, patient safety and possible health risks should always be closely considered when choosing alloys for dental restorations. For dental alloys, elemental release by corrosion is the main cause for patient exposure. To minimize the risk of possible biological adverse effects, it is advisable to choose alloys with the lowest release of elements [3]. The current standard for testing dental alloy corrosion is ISO 22674 [5]. The standard refers to the *in vitro* static immersion corrosion test (ISO 10271) [14]. In this test, the total amount of elements released from the exposed surface of the alloy specimens shall not exceed 200 μg/cm^2^ after a 7-d test period. The standard does not account for the differing toxic potential of the individual elements and combination of elements, only the total quantity.

Although the evaluation of released elements by using the ISO test has the benefit of being standardised, such a corrosion test does not reflect the continuously changing oral environment. In an attempt to optimize the simulation of oral conditions, an experimental dynamic corrosion test method was introduced. The results from the dynamic corrosion test showed less total release of elements for Aurolite 2B and Wirobond 280 along with only a slight increase for d.Sign 30. This may indicate that the repeated deflection of the alloys did not increase the level of released elements compared with static corrosion test. However, the test design only accounts for deflection, which may be insufficient to simulate occlusal wear or rubbing that could increase elemental release. A further limitation is that a 7-d test period was not enough time to alter the microstructural characteristics of the surface from stress corrosion. Since the values of the total released elements detected are low in both tests, the risk for deviations increases as a result of methodological factors. Relatively large standard deviations in these values (Table 3) illustrates this aspect.

In general, Co–Cr alloys show good corrosion resistance due to the formation of a passive oxide layer [19]. However, the passivation layer may be worn away during normal use, thereby permitting the release of elements. In Pd–Ag alloys, palladium is the main contributor to corrosion resistance as a noble element. In these alloys, the less noble elements such as Zn, Sn and In has a greater tendency to be released, which these results also confirm. Both In and Sn are oxidizing elements that migrate to the alloy surface with intention to bond with ceramics [8]. The Pd–Ag alloy released less than 0.01 µg/cm2 Pd-ions in both tests. Considering that the Pd content by weight is approximately 60%, Pd may seem to be the most corrosion resistant element in the alloy.

Partially veneered restorations are often preferred in clinical situations [20]. In these situations, only the alloy surfaces exposed to the oral environment is prone to corrosion. The parts of a restoration covered with ceramics becomes sealed and protected from corrosion. Therefore, it is critical that the oxide layer should be completely removed from areas that are not covered with ceramic. Also, the interior surface of a metal–ceramic restoration, comprising only of the alloy, becomes sealed with luting cement. Although marginal leakage can occur as a result from crevice corrosion [21], this is beyond the parameters of this study.

The d.Sign 30 alloy showed slightly higher corrosion resistance than Wirobond 280. The alloys differ in composition. Wirobond 280 contains Mo instead of Nb. To our knowledge, there is currently no studies describing corrosion behaviour of Nb containing Co–Cr dental alloys. A study on titanium alloys with Nb concluded that a Ti-6Al-7Nb showed the most corrosive resistant behaviour [22]. The authors propose next generation titanium alloys to contain Nb. The results may imply that these properties can be transferable to Co–Cr alloys. Improved wear characteristics are also found in Nb-containing Co–Cr alloys, which decrease wear particle abrasion [23].

The MTT description of ISO 10993-5 classify a compound to have a toxic potential when viability is reduced below 70% of control. According to this definition, our results showed that only Zn(II) and Co(II) had a cytotoxic potential on THP-1 cells at the concentrations tested. In and Cr exposure did not reduce viability below the 70% and would not be classified as cytotoxic according to ISO 10993-5. However, a significant reduction was recorded. This indicate that there are some interactions with the cells, and a cytotoxic potential of these elements cannot be completely ruled out. When interpreting MTT test results, it is important to emphasize that there are many possible limitations of this test. The MTT test only measures activity of one mitochondrial enzyme, and when carried out as described in the standard, the exposure time is limited to 24 h. Further, it is likely that homologue cell cultures in the laboratory are different from their *in vivo* counterparts. Therefore, the MTT results must only be used as indications of toxic potential, and a toxic potential cannot be ruled out although viability is above 70%. In addition, clinical concentrations (local and systemic) is not known for the elements we have investigated. Despite these limitations, our results indicate that Zn(II) and Co(II) are the elements with the highest toxic potential that are released from the alloys tested. Our results further indicate that their acute toxic potential is not increased in exposure mixtures with the other elements that are released from the alloys.

For further corrosion studies, it would be ideal to apply improved dynamic loading methods which better simulate oral conditions. In particular the evaluation of sliding movements on specimens which simulates oral lateral excursions could give additional data on intraoral corrosion. In addition, a higher sample size would have given even more reliable results on metal ion release. For toxicity assessments, an interesting approach would be studies on cell specific signalling pathways to evaluate immunological responses. Kim *et al.* [24] found that Co–Cr alloys induced both cytotoxicity and expression of inflammatory responses *via* the Nrf2 signalling pathway on human gingival fibroblasts and osteoblasts. However, to assess more relevant information on local and systemic toxicity of dental alloys, more randomized controlled trials are needed to enhance the clinical relevance of data on the biocompatibility of dental alloys. Although not tested in our experiments, allergy is another possible side effect of released elements that may lack a threshold concentration for onset. Most local adverse effects have been reported when noble and predominantly base metal alloys were combined [4]. A study on corrosion rate and the evaluation of combined exposure from elements in both noble and predominantly base metal alloys is relevant in this respect.

## Conclusion

The tested alloys showed low levels of metal ion release from both static and dynamic corrosion testing compared to the maximum release limit of 200 µg/cm^2^ after a 7 d test period as described in ISO 22674. Dynamic loading did not increase the metal ion release compared to the static corrosion test. Concentrations of 250 µM and above of Zn(II) and Co(II) showed a cytotoxic effect on THP-1 cells. Adding the other released elements to the exposure mixture did not seem to alter the cytotoxic potential of Zn(II) and Co(II).
